# PCSK9 inhibitors and osteoporosis: mendelian randomization and meta-analysis

**DOI:** 10.1186/s12891-024-07674-w

**Published:** 2024-07-16

**Authors:** Ding-Qiang Chen, Wen-Bin Xu, Ke-Yi Xiao, Zhi-Qiang Que, Jin-Yi Feng, Nai-Kun Sun, Di-Xin Cai, Gang Rui

**Affiliations:** 1 Department of Orthopedics, The First Affiliated Hospital of Xiamen University, Xiamen, China; 2 The School of Clinical Medicine, Fujian Medical University, Fuzhou, China; 3 Department of Orthopedics, School of Medicine, The First Affiliated Hospital of Xiamen University, Xiamen University, Xiamen, China

**Keywords:** Mendelian randomization, Osteoporosis, PCSK9, HMGCR

## Abstract

**Background:**

Proprotein convertase subtilisin/kexin type 9 (PCSK9) inhibitors represent an effective strategy for reducing cardiovascular disease risk. Yet, PCSK9’s impact on osteoporosis remains unclear. Hence, we employed Mendelian randomization (MR) analysis for examining PCSK9 inhibitor effects on osteoporosis.

**Methods:**

Single nucleotide polymorphisms (SNPs) for 3-hydroxy-3-methylglutaryl cofactor A reductase (HMGCR) and PCSK9 were gathered from available online databases for European pedigrees. Four osteoporosis-related genome-wide association studies (GWAS) data served as the main outcomes, and coronary artery disease (CAD) as a positive control for drug-targeted MR analyses. The results of MR analyses examined by sensitivity analyses were incorporated into a meta-analysis for examining causality between PCSK9 and HMGCR inhibitors and osteoporosis.

**Results:**

The meta-analysis involving a total of 1,263,102 subjects, showed that PCSK9 inhibitors can increase osteoporosis risk (*P* < 0.05, I^2^, 39%). However, HMGCR inhibitors are not associated with osteoporosis risk. Additionally, a replication of the analysis was conducted with another exposure-related GWAS dataset, which led to similar conclusions.

**Conclusion:**

PCSK9 inhibitors increase osteoporosis risk. However, HMGCR inhibitors are unremarkably linked to osteoporosis.

**Supplementary Information:**

The online version contains supplementary material available at 10.1186/s12891-024-07674-w.

## Introduction

Osteoporosis as a progressive bone disorder commonly results in a decrease in bone mass as well as microstructural deterioration of the bone, increasing bone fragility and raising the risk of fracture [1]. The pathogenesis of osteoporosis is multifaceted, with age being the predominant risk factor [2]. The rising number of aging populations worldwide and the increasing incidence of osteoporosis is now a serious international public health problem and pose great challenges to society [3]. Some studies have found that increased levels of total cholesterol and low-density lipoprotein cholesterol (LDL-C) concentrations are associated with decreased bone mass and higher fracture risk [4]. In addition, studies have identified the benefits of controlling blood lipids in lowering osteoporosis risk [5]. Thus, there is probably a correlation between the onset and development of osteoporosis and dyslipidemia, however, further research is warranted on the influence of a variety of anti-lipid agents on osteoporosis. Moreover, extensive concern has been paid to the pathogenesis of osteoporosis, and one of the most notable is the effect of inflammatory factors. Several studies have discovered that elevated levels of a number of inflammatory factors, for example, IL-1, TNF-α, and IL-6, are found in the pathogenesis of osteoporosis.

PCSK9 inhibitors, a novel class of anti-lipid agents that focus on the proprotein convertase subtilisin/kexin 9, could dramatically lower LDL-C levels [6]. PCSK9 inhibitors are now recommended for cardiovascular disease (CVD), but the impact of PCSK9 on osteoporosis remains uncertain. PCSK9i was effective in reducing Apo B, lipoprotein (a), and non-HDL cholesterol levels in addition to LDL cholesterol levels [7, 8]. Based on laboratory and clinical trials, it has been shown that hyperlipidemia affects the function of osteoblasts and upregulates osteoclast number, which in turn destroys the bone’s microscopic structure and reduces bone strength [9]. PCSK9 has also been reported to induce the expression of pro-inflammatory factors like TNF-α, IL-1β, or IL-6 [10]. Mechanisms related to the improvement of hyperlipidemia and inflammatory response by PCSK9 inhibitors may underlie their bone-protective effects. Furthermore, PCSK9 inhibitors have a low risk of side effects, such as a low incidence of adverse events like myalgia and muscle damage. However, some injection site pain or reactions may occur [11]. It is worth noting that PCSK9 inhibitors are usually used as a complementary option to lipid-lowering therapy and are mostly used clinically in conjunction with other antihyperlipidemic agents like statins. Nowadays, with no significant side effects, two antibody-based PCSK9 inhibitors are currently being successfully brought into clinics. These drugs have been demonstrated to be beneficial in lowering cholesterol and lowering the risk of events related to atherosclerotic cardiovascular disease, such as myocardial infarction, stroke, and death [12]. Therefore, PCSK9i, as a novel drug, may have a unique mechanism of action, such as indirectly affecting bone metabolism by lowering LDL levels in the blood, which may be different from the mechanism of action of traditional osteoporosis therapeutic drugs. In addition, PCSK9i has shown good safety and tolerability in clinical trials, which is one reason why it was chosen as a research treatment alternative. Most importantly osteoporosis tends to be present in the elderly population, which is often comorbid with other chronic conditions such as CAD, and this group of people who do not respond well to or are intolerant of existing osteoporosis therapeutic agents, PCSK9i may offer an alternative therapeutic option for these patients. Consequently, research on the correlation between PCSK9i and osteoporosis is very crucial. HMGCR inhibitors represent a popular family of medications used to decrease cholesterol. It has been shown that these medications may considerably lower mortality in CAD patients by up to 30%. Currently utilised in clinical practice, HMGCR inhibitors are a member of the statin medication class [13, 14]. Increases in bone mineral density (BMD) have been shown in a number of randomised controlled trials (RCTs) after statin treatment [15, 16]. The findings of many in vivo trials whereby statins accelerated bone formation suggest that these results might represent a direct impact of statins on BMD [17–19]. Another suggestion exists that LDL-C’s effects on bone metabolism influence, at least partially, the link between statin usage and BMD [20, 21]. However the relationship between HMGCR inhibitors and osteoporosis has not been adequately studied.

Drug targeting MR analysis is a novel research design in which regression analyses by modeling genetic variation in drug genetic targets’ pharmacological inhibitory effects as tool variables. This facilitates the possibility of clarifying the impacts of prolonged drug use, reinforcing the causative relationship of these pharmacogenetic targets on the potential impact of disease, and further facilitating drug repurposing, searching for new therapeutic targets, and revealing drug harms [22]. In our study, we collected relevant GWAS pooled data for examining the causality between PCSK9 and osteoporosis by drug-targeted MR analysis.

## Materials and methods

### Study design

**Fig. 1:**
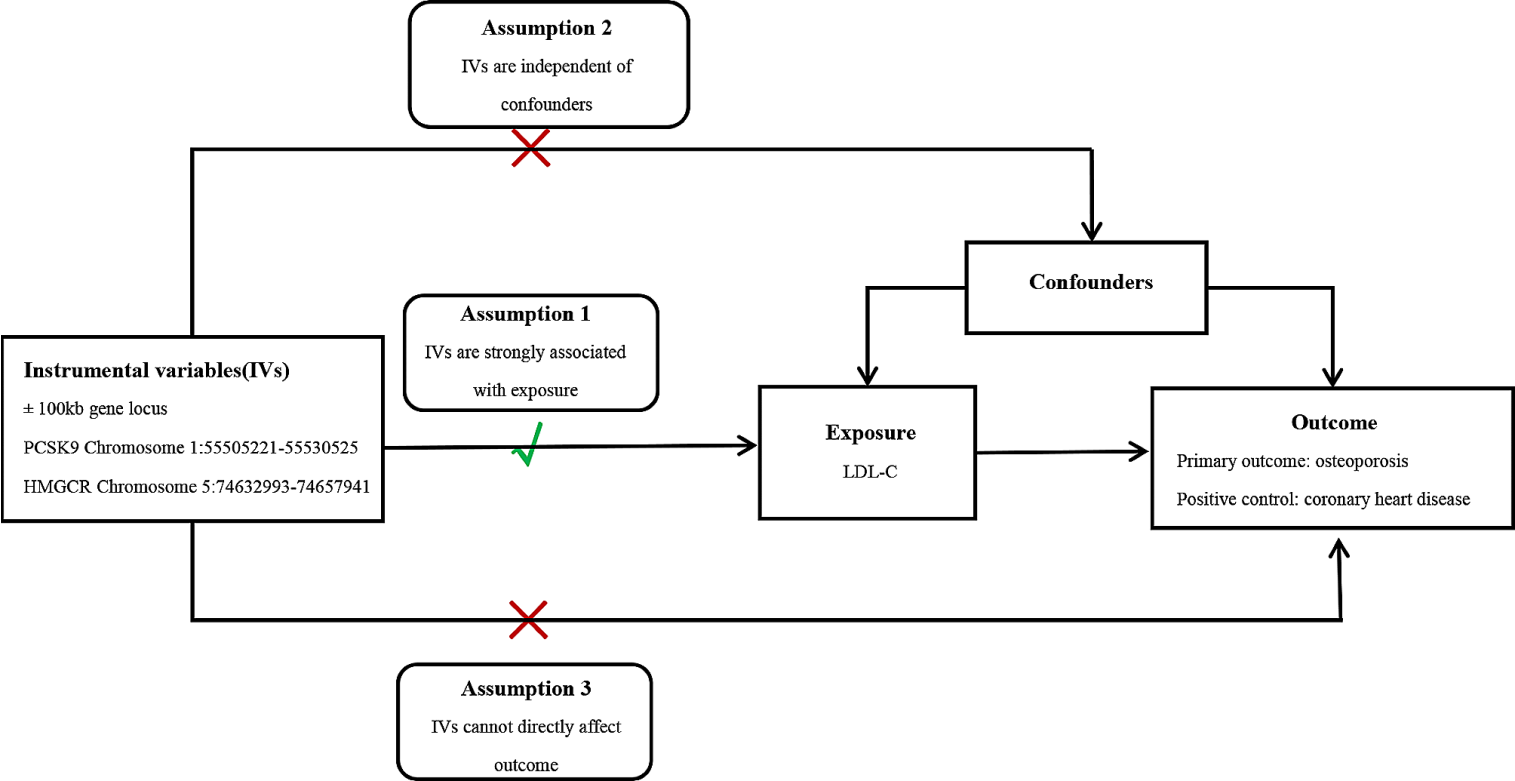
MR study design conceptual diagram

PCSK9 inhibitors are well-documented for lowering the coronary heart disease (CHD) risk. Thus, we utilised CHD pooled GWAS data as a positive control to ensure the instrumental factors’ dependability for our findings. Three assumptions should be met by genetic instruments in MR: that the exposure and the SNP are related; that the SNP is not associated with confounders that may interfere with causality between exposure and outcome; and that SNPs only display correlation with the outcome through the exposure (Fig. 1).

### Selection of PCSK9 and HMGCR instrumental variables (IVs)

GWAS pooled data for LDL-C was downloaded from the IEU Open GWAS database, containing 201,678 individuals of European origin. After acquiring IVs targeting PCSK9 and HMGCR for lowering LDL-C, they could mimic the influence of PCSK9 and HMGCR inhibitors. SNPs situated within ± 100 kb of the PCSK9 and HMGCR loci and relevant to LDL-C levels (association p-value < 5 × 10^− 8^) were chosen as IVs. In order to mitigate the impact of severe linkage disequilibrium (LD), a critical value for LD was fixed (r^2^ < 0.3). In addition, to prevent weak instrumental bias, we only used SNPs with F-statistics greater than 10. Ultimately, SNPs that fulfilled the requirements for PCSK9 and HMGCR were kept (Table S1). In addition, we used LDL-C-related exposure data from different sources derived from the circulating lipoprotein lipids and lipoprotein-related GWAS conducted by Tom G. Richardson’s team in the UK Biobank (UKBB) (comprising 440,546 individuals of European descent) [23]. Similarly, IVs of PCSK9 and HMGCR were obtained from this exposure data for drug-targeted MR analysis using the methods described above (Table S2). By repeating the analysis in multiple independent samples, we could verify the consistency of the results and enhance the confidence of the findings. The different datasets represent different populations and geographic regions, which helps us to assess the generalizability of the results. In addition there may be genetic and environmental differences across populations, and using multiple datasets can help us explore the impact of these differences on the results.

### Source of outcomes

The primary outcome of the drug target MR analysis was osteoporosis, with CHD selected as the positive control. These datasets were studied in populations from Europe. The CHD data was derived from the GWAS pooled data, which contains 21,012 cases and 197,780 controls. In addition, we also collected pooled data on osteoporosis from four different GWAS studies (Table 1).

**Table 1:** Summary of GWAS datasets included in this study

Phenotype	Sample size (case/control)	Number of SNPs	Population	Units
Exposure				
LDL-C (ieu-b-5089)	2,01,678	1,23,21,875	European	SD
LDL-C (ieu-b-110)	440546	12321875	European	NA
Positive control				
CHD (finn-b-I9_CHD)	218792(21,012/197,780)	16380466	European	NA
Outcomes				
Osteoporosis (ukb-b-12141)	462,933(7,547/455,386)	98,51,867	European	SD
Osteoporosis (ukb-b-17796)	463,010(1,976/461,034)	9851867	European	SD
Osteoporosis (ukb-a-87)	337,159(5,266/331,893)	10894596	European	SD
Osteoporosis (finn-b-M13_OSTEOPOROSIS)	212778(3,203/209,575)	16380452	European	NA

SNP, single nucleotide polymorphism; CHD, coronary heart disease

### Data analysis

The main analysis method adopted was the IVW approach. Four other methods were used for validation: MR Egger, weighted median, simple mode, and weighted mode.

The MR Egger approach and the IVW approach were utilized for detecting heterogeneity. The heterogeneity of genetic tools was assessed according to Cochrane’s Q-value, and *P* > 0.05 indicated no meaningful heterogeneity. In addition, using the MR Egger regression equation, the horizontal pleiotropy of the genetic instruments was evaluated; a value of *P* > 0.05 indicated that no horizontal pleiotropy.

To make sure that SNPs had nothing to do with the outcomes, we also searched for characteristics that were directly linked to SNPs using the PhenoScanner website (http://www.phenoscanner.medschl.cam.ac.uk/). After removing outliers from the MR-Egger regression and MR-pleiotropy residual sum outlier test, sensitivity analysis was again carried out. To guarantee that a particular SNP did not significantly impact the outcomes, we removed each SNP in turn using the leave-one-out method for assuring stable results.

Finally, we included the results of the Mendelian randomized studies that passed the sensitivity analysis in the meta-analysis. R 4.3.1 was utilized for conducting all analyses.

## Results

### Positive control analyses

**Fig. 2:**
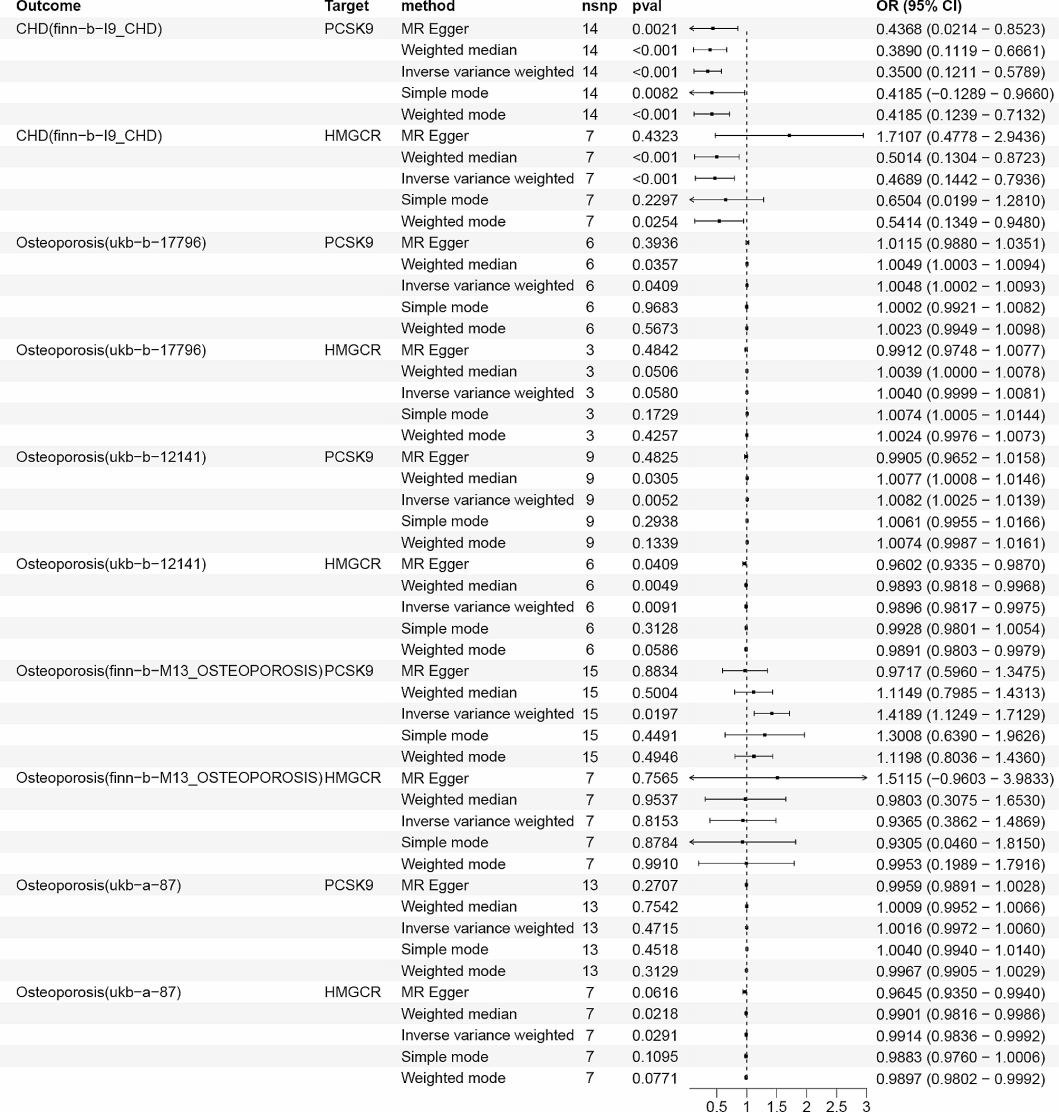
The impact of PCSK9 and HMGCR inhibitors on CHD and osteoporosis

As we have learned, the IVW approach’s findings demonstrated that PCSK9 inhibitors considerably lowered the risk of CHD (OR: 0.349, 95% CI 0.121–0.578, *P* = 2.447 × 10^− 19^). HMGCR inhibitors also showed an analogous effect (OR: 0.468, 95% CI 0.144–0.793, *P* = 4.851 × 10^− 06^). Furthermore, the outcomes from the last four MR analysis techniques were also comparable (Fig. 2 and Table S3). Results from a repeat study using a different GWAS dataset were similar (Table S4).

### The causal relationship between osteoporosis and inhibitors of PCSK9 and HMGCR

Three of the four studies analyzing data from the osteoporosis GWAS data showed that inhibition of PCSK9 increased the risk of osteoporosis. ukb-b-12,141 (OR: 1.0081, 95% CI 1.0024–1.0138, *P* = 0.0051), ukb-b-17,796 (OR: 1.0047, 95% CI 1.0002–1.0092, *P* = 0.0409), finn-b-M13_OSTEOPOROSIS (OR: 1.4188, 95% CI 1.1248–1.7128, *P* = 0.0196), ukb-a-87 (OR: 1.0016, 95% CI 0.9972–1.0059, *P* = 0.4715) (Fig. 2 and Table S3).

In addition, some of the results of the GWAS data analysis showed that genetically predicted HMGCR inhibition reduced the risk of osteoporosis. ukb-b-12,141 (OR: 0.9895, 95% CI 0.9817–0.9974, *P* = 0.0091), ukb-a-87 (OR: 0.9913, 95% CI 0.9836–0.9991, *P* = 0.0291), finn-b-M13_OSTEOPOROSIS (OR: 0.9365, 95% CI 0.3861–1.4868, *P* = 0.8153), ukb-b-17,796 (OR: 1.0040, 95% CI 0.9998–1.0081, *P* = 0.0579). Moreover, the outcomes of the last four MR analysis techniques were analogous (Fig. 2 and Table S3).

Moreover, we repeated the analysis using different GWAS data and came to similar results (Table S4).

### Sensitivity analysis

Sensitivity analysis suggests horizontal pleiotropy exist in MR analysis between PCSK9 inhibitors and osteoporosis (finn-b-M13_OSTEOPOROSIS). The results of this analysis were therefore rejected for inclusion in subsequent meta-analyses. In addition, there was heterogeneity in the results of individual analyses, and this heterogeneity may have stemmed from independent Mendelian variation laws rather than present pleiotropy [24] (Table S5 and Table S6). Furthermore, after excluding each single SNP, the results of the IVW analysis remained similar (Fig S1).

### Meta-analysis

**Fig. 3:**
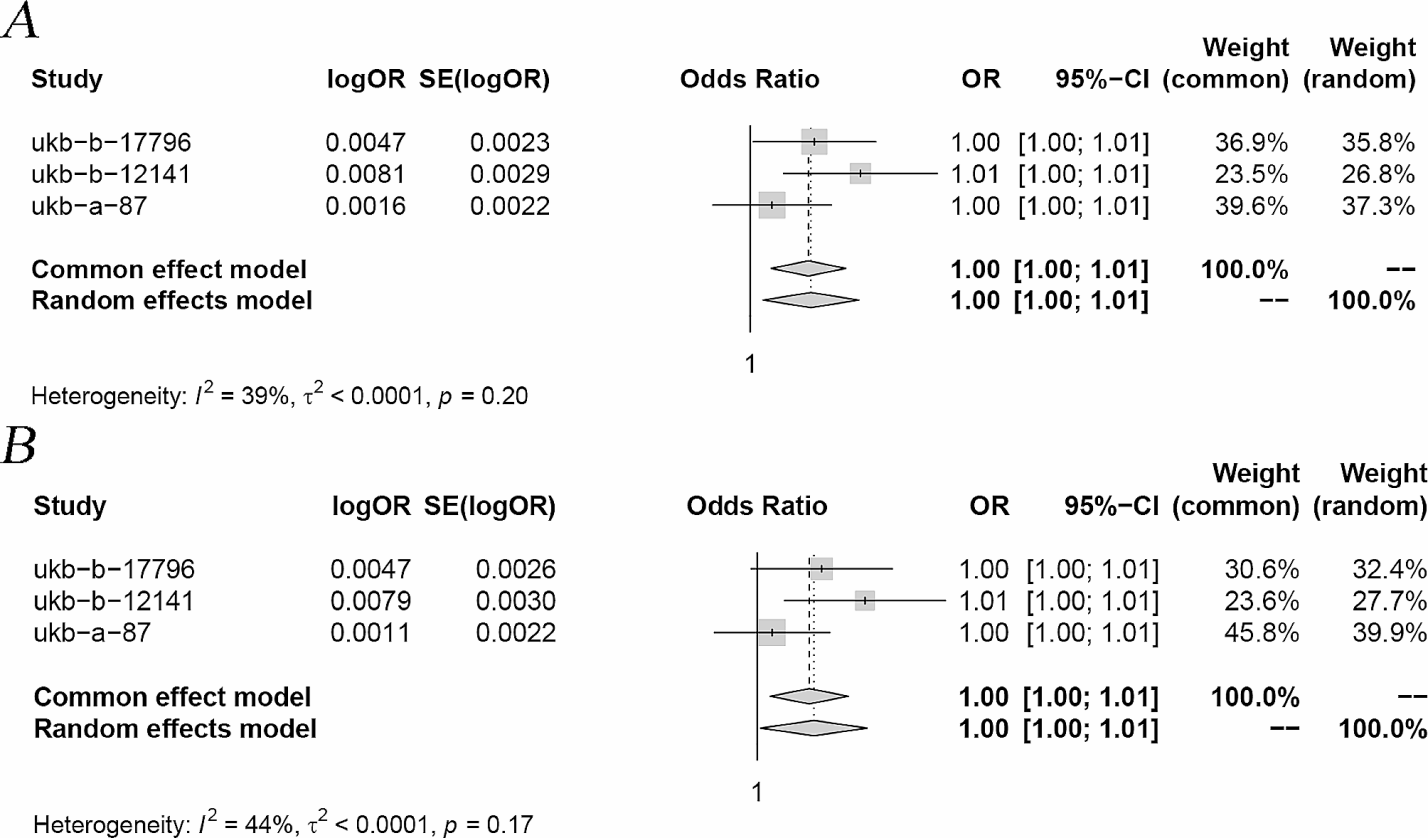
Meta-analytic forest figure showing the effect of PCSK9 inhibitors on osteoporosis. (**A**) Initial analysis; (**B**) Repeat analysis

**Fig. 4:**
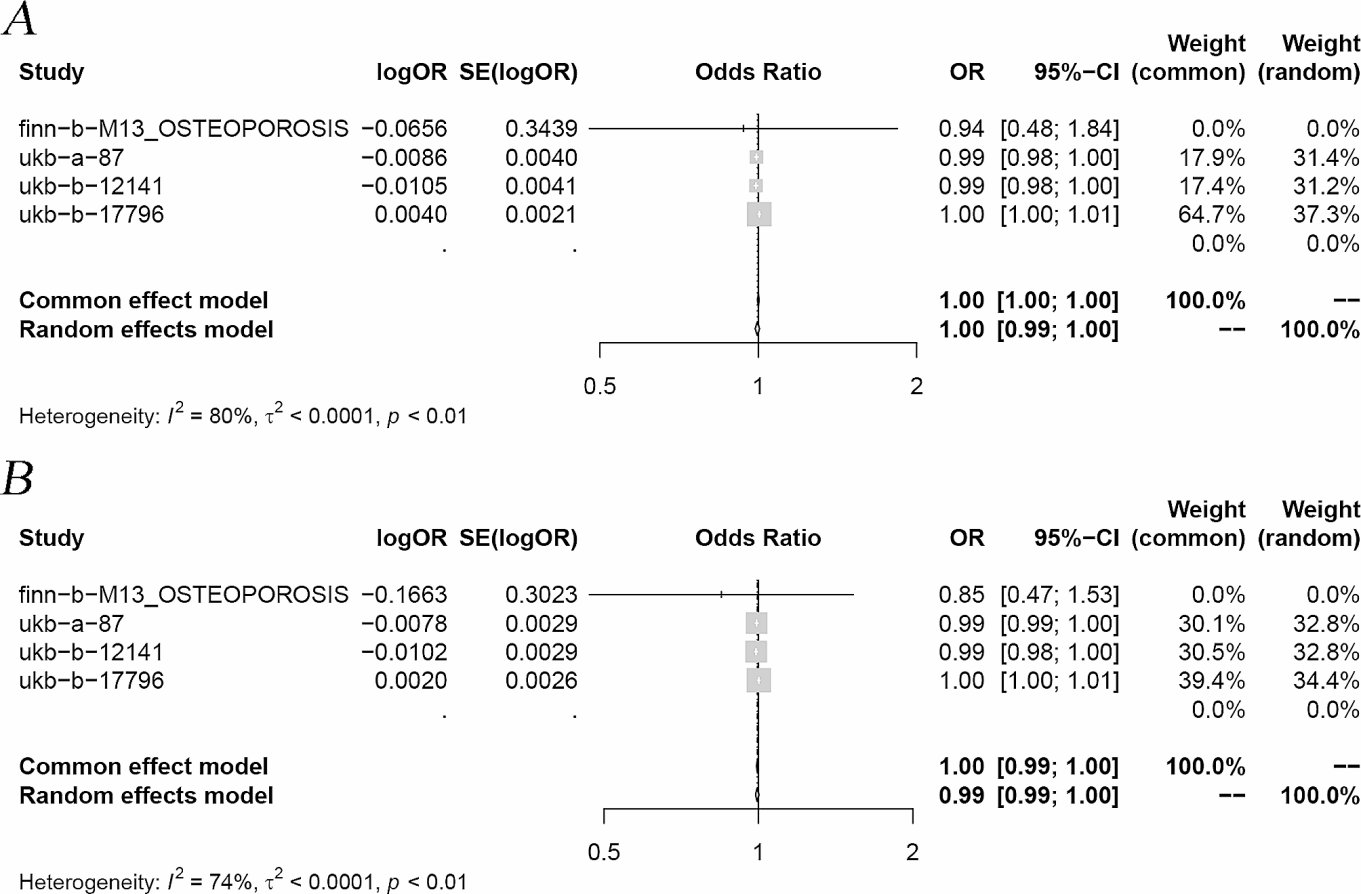
Meta-analytic forest figure showing the effect of HMGCR inhibitors on osteoporosis. (**A**) Initial analysis; (**B**) Repeat analysis

For more robust outcomes, we included in the meta-analysis the results of multiple MR analyses that passed the sensitivity analysis test. Following the meta-analysis, it was observed that PCSK9 inhibitors had an increased risk of osteoporosis (Common effects model: OR: 1.00, 95% CI 1.00-1.01 *P* < 0.05 ) (Fig. 3 ), whereas the impact of HMGCR inhibitors on osteoporosis was not significant (Random effects model: OR: 0.99, 95% CI 0.99-1.00 *P* > 0.05) (Fig. 4).

## Discussion

PCSK9i is a category of anti-lipid agents that are thought to have several beneficial effects besides their LDL-C lowering properties [25]. Latest study demonstrates that PCSK9 is produced by the macrophages, vascular endothelium, and smooth muscle cells and triggers the release of pro-inflammatory factors from macrophages, hepatocytes, and various tissues [26]. Thus inhibition of PCSK9 is very important in controlling inflammation. Osteoporosis, as a systemic bone disease, can cause pain, spinal deformity, fractures, and many other complications, affecting millions of aging people worldwide [27]. The etiology of osteoporosis is multifactorial, and the overproduction of pro-inflammatory factors has been recognized as exerting critical functions in the onset and progression of osteoporosis. It has been observed that IL-1α, IL-1β, IL-6, and TNF-α cell factors alter the balance between osteoblasts and osteoclasts [28]. It has also been shown that there is a direct link between high blood fat anemia and osteoporosis. A clinical trial found that in the fasted state, postmenopausal women whose lipid profile causes atherosclerosis have worse BMD in the lumbar spine, total hip, and femoral neck skeleton compared with normolipidemic controls [29]. There is also the “lipid hypothesis of osteoporosis” which claims that oxidized lipids are contributing factors to osteoporosis [30]. Thus PCSK9 ought to have an unforeseen function in osteoporosis. However, the link between PCSK9 and osteoporosis has not yet been thoroughly investigated.

In this MR analysis, we chose important SNPs near PCSK9, which encodes the proprotein convertase subtilisin/kexin type 9, to mimic the agonistic effects of the PCSK9. However, contrary to our expectations that these SNPs failed to reduce the risk of osteoporosis and instead increased the chances of osteoporosis, these findings will help to achieve a deeper understanding of PCSK9’s effects and provide clues to the possible side effects of PCSK9.

In the human body, the growth and upkeep of bones are significantly influenced by the hormones androgens and oestrogens. One of the risk factors for osteoporosis is decreased oestrogen production following menopause in women and decreased oestrogen and androgen production in elderly men [31]. It can be argued that decreased estrogen represents a critical step in the pathogenesis of degenerative osteoporosis [32]. PCSK9 inhibitors, an emerging category of anti-lipid agents, are effective in lowering plasma LDL-C levels by inhibiting the LDL acceptors degradation [33]. However, estrogen as a derivative of cholesterol exerts an essential effect on bone metabolism through the inhibition of bone resorption [34]. In both mouse models and cell lines, statins can decrease testosterone, estradiol, and luteinizing hormone levels in plasma, while raising follicle-stimulating hormone and luteinizing hormone levels [35, 36]. In addition, a cross-sectional study based on 4,166 male participants showed statins were linked to markedly reduced serum levels of total and non-SHBG-bound testosterone [37]. In addition, statins may exert an anabolic effect via blocking the reductase of 3-hydroxy-3-methylglutaryl coenzyme (HMG-CoA) of the mevalonate pathway. Isoleprenoids like farnesyl pyrophosphate and geranylgeranyl pyrophosphate, which are crucial for bone cell differentiation and bone formation, can only be synthesised by the mevalonate route [38]. Such discoveries indicate that PCSK9 inhibitors may promote the development of osteoporosis by modulating estrogen levels. A large number of clinical trials are needed for future validation.

For comparison, it was shown that HMGCRi did not raise the risk of osteoporosis, and some of the individual GWAS data analyzed suggested that HMGCR inhibitors decreased osteoporosis risk. This is the same ending as we had previously envisioned, that lipid-lowering drugs can reduce the risk of osteoporosis by modulating LDL-C levels and thereby regulating the number and function of osteoblasts and osteoclasts, improving bone microarchitecture, and increasing bone strength.

Nonetheless, this research has many inevitable drawbacks. First, MR analysis is only a method of data analysis based on an online database and needs to be validated in the objective world through lots and plenty of clinical trials. Second, the target population was mainly European. The results of the study have limited application to non-European populations and specific genders. GWAS data from other ethnic populations could be incorporated into future studies to enhance the scope of applicability of the findings. Third, the meta-analysis section of this study contains some heterogeneity, which may stem from the different databases from which we sourced the data included in the analysis. This is manifested in the following ways: different databases may use different data collection methods, which may lead to differences in data quality and consistency. Also, different databases may contain studies with different designs, such as RCTs, observational studies, etc. These differences in study design may lead to heterogeneity of results. In addition, data from different populations, including age, gender, ethnicity, socio-economic status, and so on. Differences in the characteristics of these populations may affect the results. Studies in different databases may also use different outcome measurement tools or assessment criteria, which may lead to heterogeneity of results. In addition, some databases may tend to include studies with significant outcomes, which may lead to publication bias and thus affect the homogeneity of the meta-analysis. Different databases may cover studies from different time periods, and treatments, diagnostic criteria, etc. may change over time, which may affect the homogeneity of results. Studies in different regions may be affected by geographic and cultural factors, which may manifest in data from different databases, leading to heterogeneity. Data completeness and accuracy may also vary across databases; some databases may contain more detailed data, while others may contain more abbreviated data. At the same time, different studies may use different statistical methods to analyze the data, which may lead to inconsistent results even if the study designs are similar [39]. The presence of heterogeneity can lead to reduced reliability of meta-analysis results, limited generalizability of conclusions, compromised policy and clinical decisions, impaired study validity, and publication bias [40]. Therefore, we used the data shown in the random effects model as the results of the meta-analysis to minimize heterogeneity. We reiterate that despite the heterogeneity, our study has important implications for the treatment and prevention of osteoporosis and provides new insights into medication choices for patients with CAD and other osteoporotic conditions requiring lipid-lowering therapy. Finally, MR studies are an epidemiological methodology that utilizes genetic variation as a natural experiment to assess the causality between exposure factors (such as drugs, diet, or lifestyle) and disease. Although this approach can be very useful in some situations, it does have some limitations compared to RCTs; MR studies rely on specific genetic variants as IVs that must satisfy the conditions of being strongly associated with the exposure factor, independently associated with the disease, and unaffected by confounding factors. If these conditions are not met, the results of the study may be biased. Genetic variants may affect multiple biological pathways, not just the exposure factor of interest in the study. This pleiotropy may lead to mischaracterization of causality. MR studies usually focus on common genetic variants, whereas rare variants may have an important effect on disease risk, but these variants may be overlooked in MR studies. MR studies may need extremely high sample sizes to discover links between genetic variations and illness, which may be challenging to obtain in certain situations. In addition even if genetic variants are strongly associated with exposure factors, they may influence disease risk through other unconsidered pathways, and such spillover effects may lead to misinterpretation of causality. MR studies are usually based on cross-sectional or retrospective data, which limits the determination of temporal order, an aspect that can be better controlled by RCTs. Interactions between genetic variation and environmental factors may influence the relationship between exposure factors and disease, and MR studies may not adequately capture such interactions. The results of MR studies may not be generalizable because genetic effects may vary across populations or environments. MR studies rely on existing genetic data, which may be subject to selectivity bias or information bias [41, 42]. It’s possible that the outcomes of MR research won’t immediately apply to the creation of clinical practice recommendations or regulations because they are usually based on observational data rather than interventional studies like RCTs. Despite these limitations, MR studies provide a powerful tool for assessing causality when RCTs are not possible or are too costly. Researchers need to be cautious when interpreting the results of MR studies and consider the impact of these limitations on the findings. It is worth noting that three of our MR studies were based on data from UKB. Due to the extensive participation in UKB and the multifaceted study design, there was some degree of participant overlap. This overlap was largely due to the fact that participants may have been eligible for more than one study and therefore included in different research programs. There may be some limitations on the ability to generalize the results of our study. However, although these studies were from the same database, they differed in sample selection, study design, and analytic methods. We chose these studies because they provided a large amount of representative data that helped to improve the statistical power of our analyses and allowed us to explore the association between PCSK9 and osteoporosis risk in different subgroups. We recognize that data overlap may impose some limitations on the interpretation of results. However, we believe that the methodological and analytic diversity of these studies provides complementary perspectives for assessing the impact of PCSK9. In addition, these GWAS studies offer significant advantages in terms of scientific contribution, sample size, and study quality. For these reasons, we believe that the selection of these studies for MR analysis was justified. Future studies could consider more detailed analyses of participants to further address overlap and provide deeper insights.

## Conclusion

With this drug-targeted MR analysis, genetic variation within the PCSK9 gene is associated with osteoporosis risk suggesting PCSK9 inhibitors might increase this risk, whereas HMGCR inhibitors were not associated with osteoporosis. More in-depth investigations are necessary in the future to discover the underlying mechanisms.

### Electronic supplementary material

Below is the link to the electronic supplementary material.

Supplementary Material 1

Supplementary Material 2

Supplementary Material 3

Supplementary Material 4

Supplementary Material 5

Supplementary Material 6

Supplementary Material 7

## Data Availability

All data are available from open online databases including IEU Open GWAS database (https://gwas.mrcieu.ac.uk/) and National Library of Medicine (https://www.ncbi.nlm.nih.gov/).
