# Supplementary material for: PCSK9 inhibitors and osteoporosis: mendelian randomization and meta-analysis
Source: BMC Musculoskelet Disord. 2024 Jul 16;25:548. doi: 10.1186/s12891-024-07674-w (PMC11251371; doi:10.1186/s12891-024-07674-w)
Supplement: Supplementary file 7 — Supplementary Material 7 [file 12891_2024_7674_MOESM7_ESM.docx]

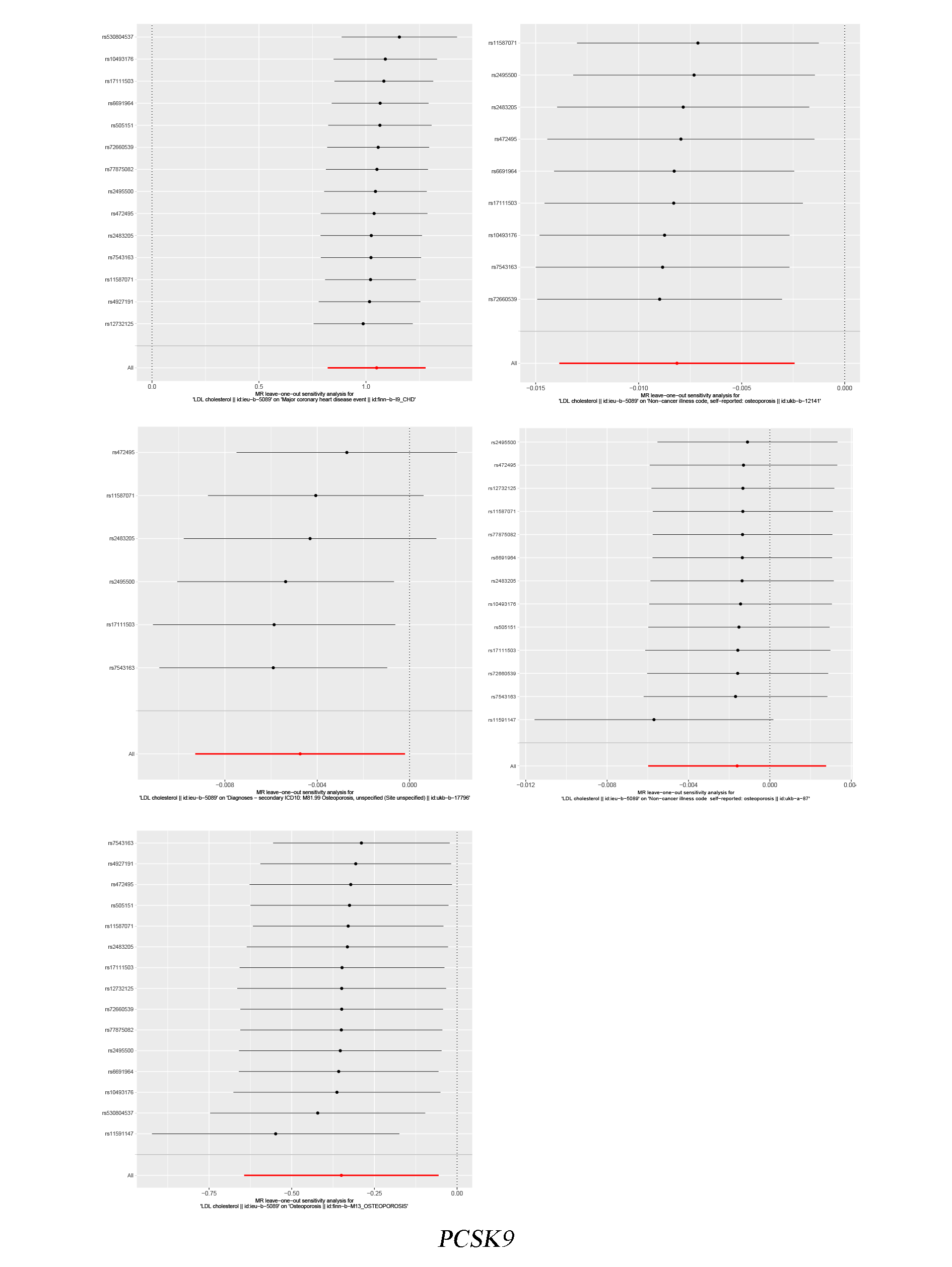


Leave-one-out analysis analysis of PCSK9 on coronary heart disease and osteoporosis. The leave-one-out method is used to evaluate the excessive impact of a single SNP on MR analysis if the comprehensive effect of the remaining SNPs is consistent with the main effect after removing one SNP. SNP, single nucleotide polymorphisms; PCSK9, proprotein convertase subtilisin/kexin type 9;


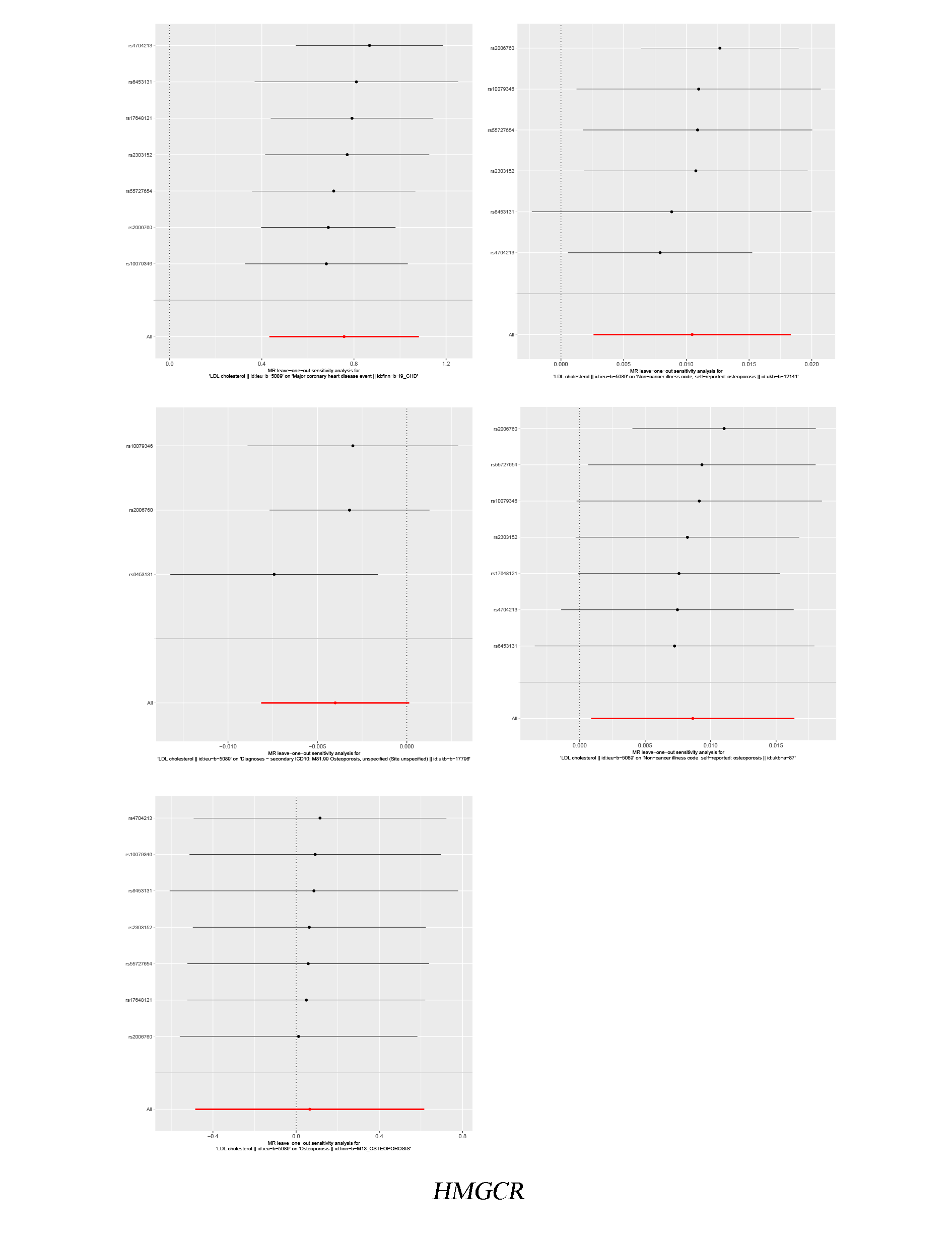


Leave-one-out analysis analysis of HMGCR on coronary heart disease and osteoporosis. The leave-one-out method is used to evaluate the excessive impact of a single SNP on MR analysis if the comprehensive effect of the remaining SNPs is consistent with the main effect after removing one SNP. SNP, single nucleotide polymorphisms; HMGCR, 3-hydroxy-3-methylglutaryl-coenzyme A reductase;


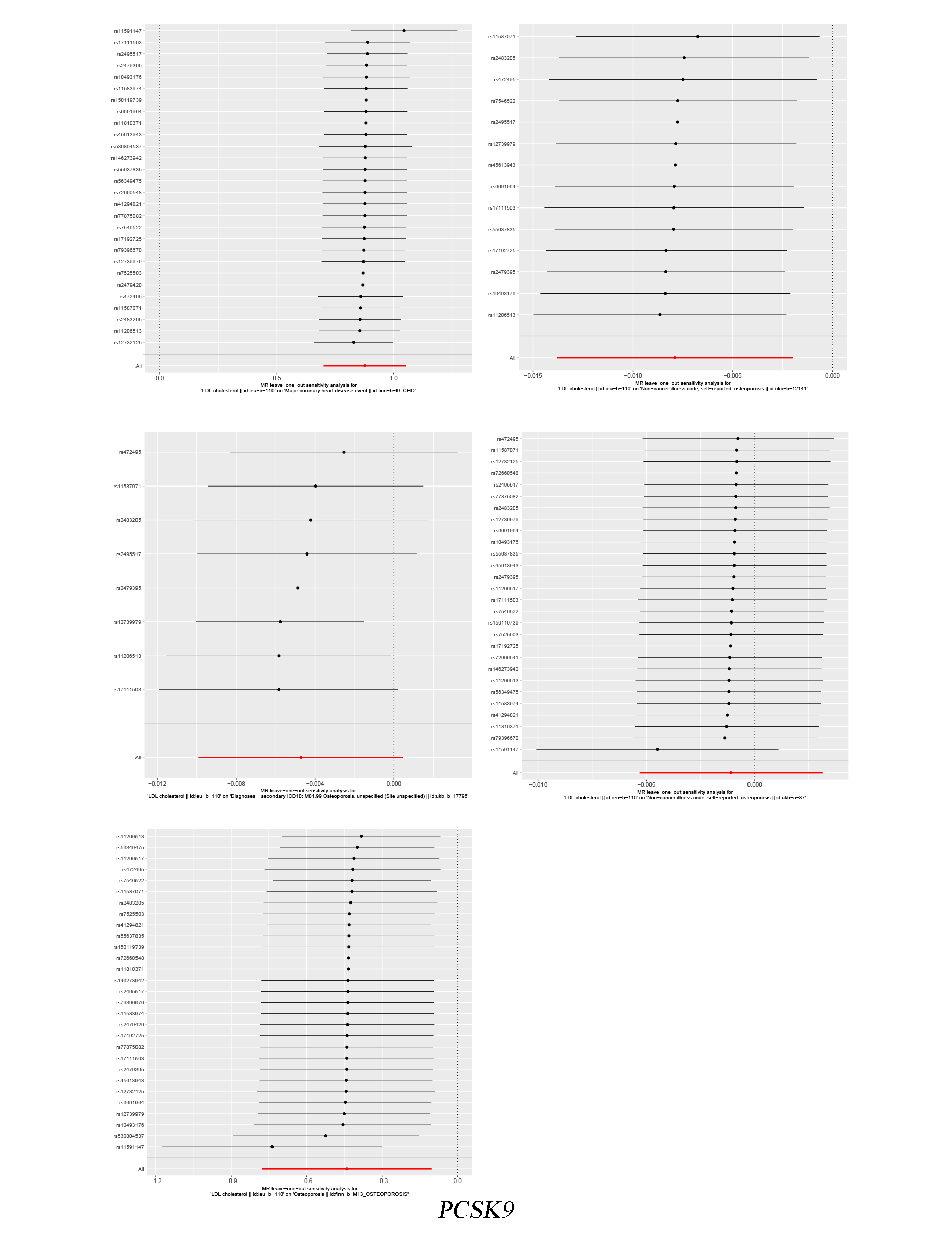


Leave-one-out analysis analysis of PCSK9 on coronary heart disease and osteoporosis in the repeated analysis. The leave-one-out method is used to evaluate the excessive impact of a single SNP on MR analysis if the comprehensive effect of the remaining SNPs is consistent with the main effect after removing one SNP. SNP, single nucleotide polymorphisms; PCSK9, proprotein convertase subtilisin/kexin type 9;


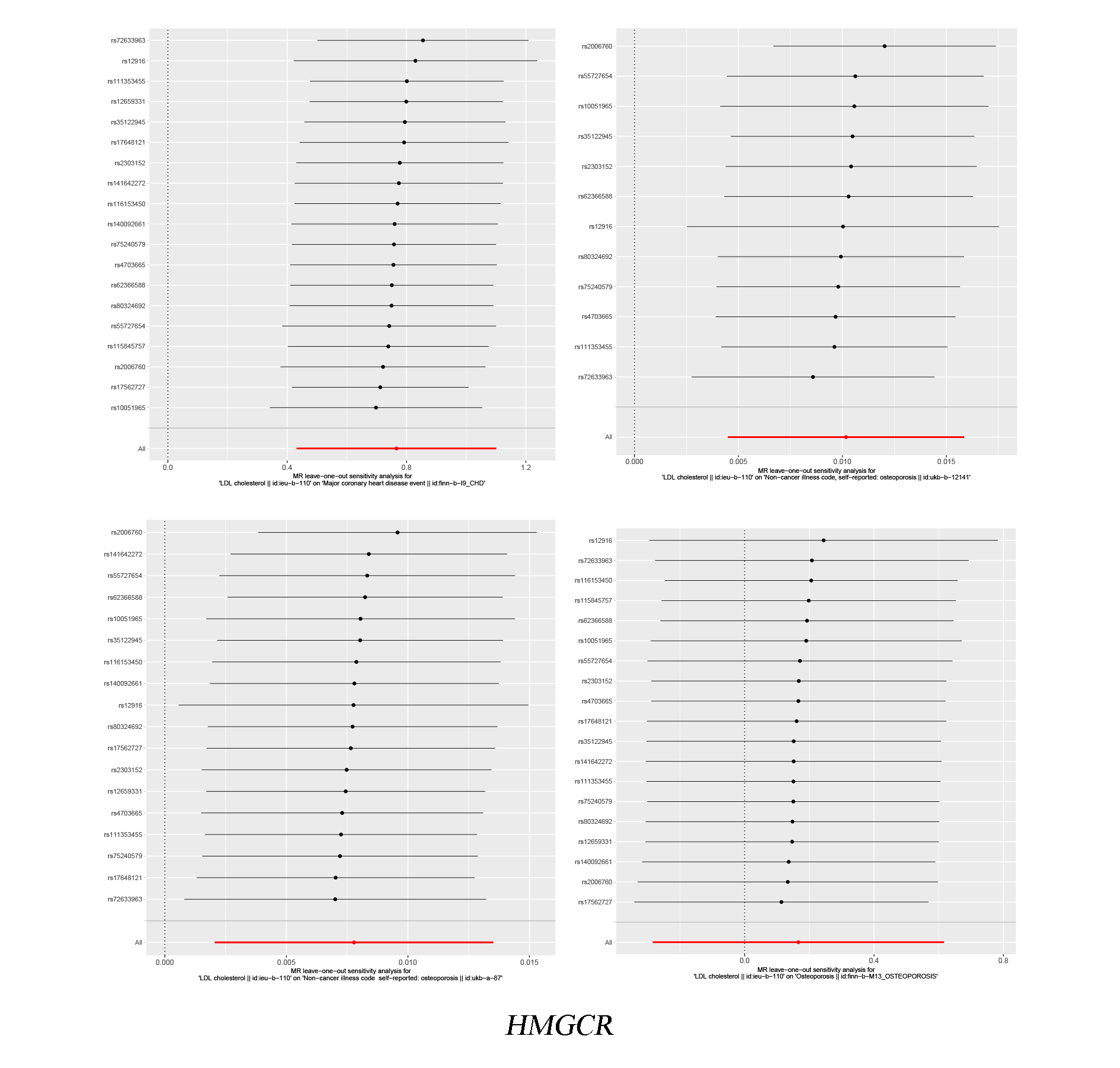


Leave-one-out analysis analysis of HMGCR on coronary heart disease and osteoporosis in the repeated analysis. The leave-one-out method is used to evaluate the excessive impact of a single SNP on MR analysis if the comprehensive effect of the remaining SNPs is consistent with the main effect after removing one SNP. SNP, single nucleotide polymorphisms; HMGCR, 3-hydroxy-3-methylglutaryl-coenzyme A reductase;
